# Long-Term Clinical Trajectory of Patients with Subarachnoid Hemorrhage: Linking Acute Care and Neurorehabilitation

**DOI:** 10.1007/s12028-022-01572-6

**Published:** 2022-08-12

**Authors:** Anna Lindner, Luca Brunelli, Verena Rass, Bogdan-Andrei Ianosi, Max Gaasch, Mario Kofler, Victoria Limmert, Alois J. Schiefecker, Bettina Pfausler, Ronny Beer, Elke Pucks-Faes, Raimund Helbok

**Affiliations:** 1 Neurological Intensive Care Unit, Department of Neurology, Medical University of Innsbruck, Anichstrasse 35, 6020 Innsbruck, Austria; 2 Institute of Medical Informatics, UMIT: University for Health Sciences, Medical Informatics and Technology, Eduard Wallnoefer-Zentrum 1, 6060 Hall in Tirol, Austria; 3 Department of Neurology, Hochzirl Hospital, Hochzirl 1, 6170 Zirl, Austria

**Keywords:** Subarachnoid hemorrhage, Neurorehabilitation, Critical care

## Abstract

**Background:**

Despite improvements in the critical care management of subarachnoid hemorrhage (SAH), a substantial number of patients still suffer from disabilities. In most areas of the world, longitudinal follow-up is not routinely performed, and the patient’s trajectory remains unknown.

**Methods:**

We prospectively collected data of 298 consecutive patients with spontaneous SAH and evaluated clinical trajectories at discharge, 3 months, and 1 year after SAH. In a subgroup of patients transferred to a local neurorehabilitation center (Rehab-Hochzirl), we studied the effects of rehabilitation intensity on clinical trajectories. Any decrease in the modified Rankin Scale (mRS) was defined as an improvement, with mRS ≤ 2 indicating good outcome. We used multivariate generalized linear models to investigate associations with clinical trajectories.

**Results:**

Out of the 250 surviving patients, 35% were transferred directly to Rehab-Hochzirl (*n* = 87 of 250; mRS at discharge = 4), 11% were transferred to another rehabilitation center (*n* = 27 of 250; mRS = 1), 1% were transferred to a nursing home (*n* = 3 of 250; mRS = 5), 21% were transferred to their country of origin (*n* = 52 of 250; mRS = 4), and 32% (*n* = 79 of 250; mRS = 1) were discharged home. Functional outcome improved in 57% (*n* = 122 of 215) of patients during the first 3 months, with an additional 16% (35 of 215) improving between 3 and 12 months, resulting in an overall improvement in 73% (*n* = 157 of 215) of survivors. After 1 year, 60% (*n* = 179 of 250) of patients were functionally independent. A lower Hunt and Hess scale score at intensive care unit admission, younger age, a lower mRS at intensive care unit discharge, fewer days on mechanical ventilation, and male sex were independently associated with better functional recovery. Although the subgroup of patients transferred to Rehab-Hochzirl were more severely affected, 60% (52 of 87) improved during inpatient neurorehabilitation.

**Conclusions:**

Our results indicate ongoing functional improvement in a substantial number of patients with SAH throughout a follow-up period of 12 months. This effect was also observed in patients with severe disability receiving inpatient neurorehabilitation.

**Supplementary Information:**

The online version contains supplementary material available at 10.1007/s12028-022-01572-6.

## Introduction

With an overall mortality rate of up to 50% and a high prevalence of functional impairment, spontaneous subarachnoid hemorrhage (SAH) contributes significantly to stroke-related loss of productive life years, especially due to the young age of patients with SAH [1, 2]. Improvements in critical care management led to increased numbers of survivors [3, 4], which in turn resulted in a higher number of patients with functional and cognitive impairment and restrictions in quality of life [5]. The care of patients with SAH after discharge from the intensive care unit (ICU) is becoming increasingly important. Nevertheless, little data exist on clinical trajectories and long-term outcome in a diversity of clinical settings [6, 7]. Neurorehabilitation supports recovery [8, 9], however, access to specialized rehabilitation facilities differs around the world and follow-up is not performed in most centers. Subsequently, there is a knowledge gap about clinical trajectories and how neurorehabilitation impacts the patient’s condition, especially in those with severe disability after ICU discharge.

In this study we aimed to (1) describe clinical trajectories over a 1-year period in consecutive patients with SAH and to (2) quantify neurorehabilitation treatment intensities in a subgroup of patients who were transferred to a rehabilitation facility linked to our center. We hypothesized that the majority of patients improve over time, including severely disabled patients transferred to inpatient neurorehabilitation.

## Methods

The data that support the findings of this study are available from the corresponding author on reasonable request.

### Patient Selection and Study Setting

**Fig. 1:**
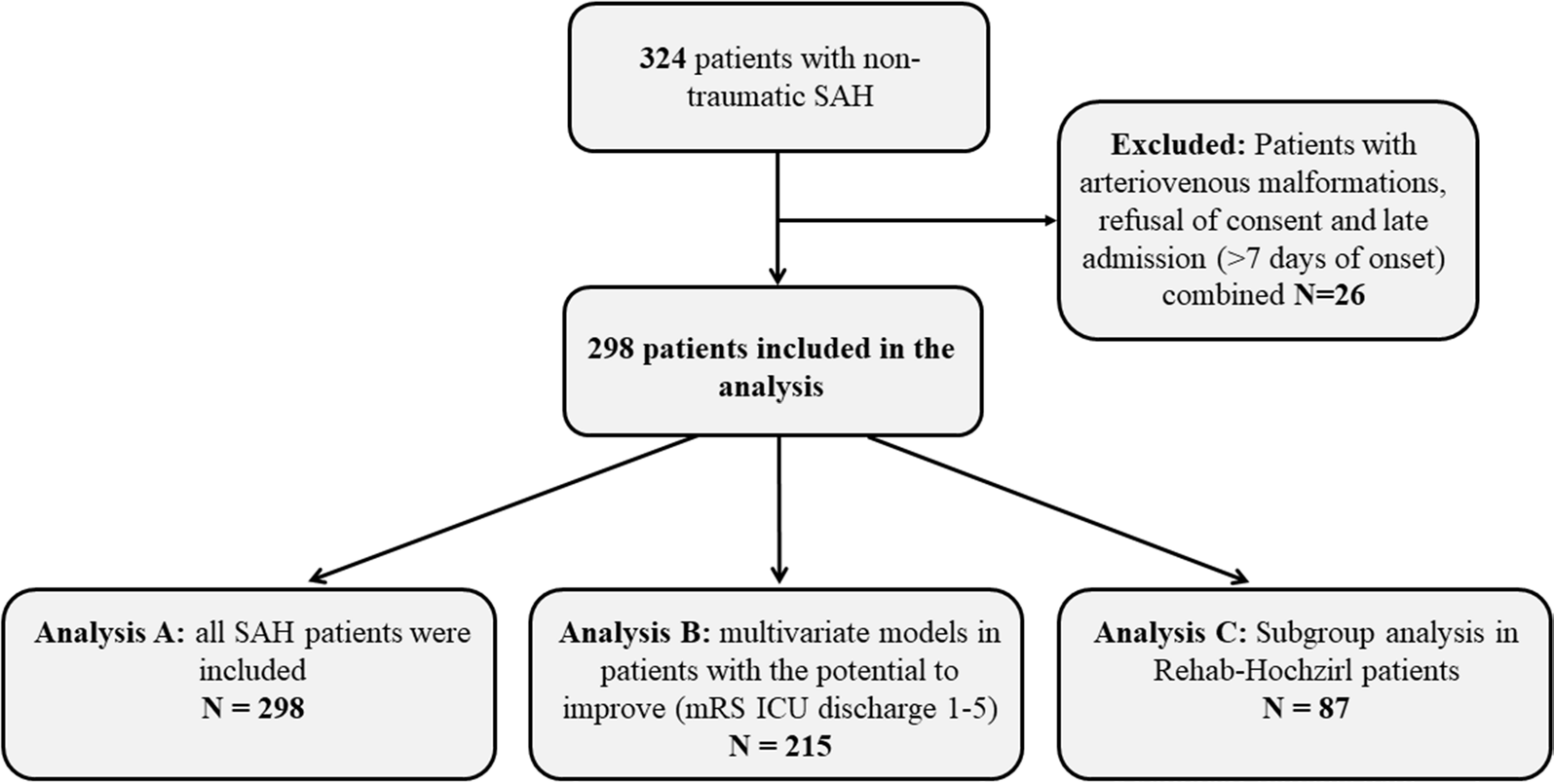
Patient selection. Flowchart showing the selection of eligible patients and the different analysis groups. *ICU* intensive care unit, *mRS* modified Rankin Scale, *SAH* subarachnoid hemorrhage

This study is a retrospective analysis of prospectively collected consecutive patients with nontraumatic SAH admitted between 2010 and 2017 to the neurological ICU of a tertiary hospital (Medical University of Innsbruck) who were prospectively included in an institutional registry. A total of 324 patients were screened for eligibility. Inclusion criteria were nontraumatic SAH, age ≥ 18 years, and ICU stay for at least 24 h. Patients with aneurysmal bleeding within arteriovenous malformations, refusal of consent, and late admission (> 7 days of onset; combined *n* = 26) were excluded, leaving 298 patients eligible for final analysis (Fig. 1).

The conduct of the study was approved by the hospital’s institutional review board (Medical University of Innsbruck, AM4091-292/4.6). Written informed consent was obtained from all patients according to local regulations, in accordance with the Declaration of Helsinki.

### Patient Management and Data Collection

Clinical management of patients with SAH conformed to current international guidelines set forth by the American Heart Association and European Stroke association [10, 11], with the exception that nimodipine was routinely administered intravenously in patients with poor-grade SAH. The Hunt and Hess scale score (H&H) [12] and the modified Fisher Score [13, 14] were used to assess the clinical and radiographic disease severity on admission. Ruptured aneurysms were treated, according to the consultation of an interdisciplinary team (consisting of neurology, neuroradiology, and neurosurgery) by either neurosurgical clipping or endovascular coiling. All patients were repeatedly screened for the development of large-vessel cerebral vasospasm, using transcranial color-coded duplex sonography (LOGIQ S8; GE Healthcare, Chicago, IL). Mean velocities > 120 cm/s in the middle or anterior cerebral artery or daily changes in mean velocities > 50 cm/s were defined as vasospasm. Patients who developed vasospasm were treated with induced hypertension targeting a cerebral perfusion pressure > 80 mmHg, with individual adjustments being made on the basis of clinical, radiographic, and multimodal neuromonitoring data. Severe refractory vasospasm (> 200 cm/s) was further confirmed by catheter cerebral panangiogram and evaluated for intraarterial nimodipine application. Delayed cerebral ischemia was defined as a clinical deterioration of ≥ 2 points on the Glasgow Coma Score or National Institutes of Health Stroke Scale, the occurrence of new focal neurological symptoms, or a new infarction on computed tomography scan without any other underlying cause to be determined [15].

Patients’ baseline characteristics, disease specific characteristics, treatment interventions, and hospital complications were prospectively recorded in our institutional SAH database and confirmed in weekly meetings of the study team and treating neurointensivists.

### Assessment of Functional Outcome at 3-Month and 12-Month Follow-Up

We evaluated clinical trajectories based on the modified Rankin Scale (mRS) over a 1-year period, with any decrease in the mRS (greater than one point) indicating improvement. Good functional outcome was defined as mRS ≤ 2. Outcomes at ICU discharge were prospectively assessed by the study team. Functional outcome after 3 months was assessed by a study nurse anonymized to the clinical course of the patient via telephone interview. After 12 months, patients were either examined in person by trained neurologists or, in case of patient nonattendance, via telephone interview.

In a subgroup of patients transferred to the neurorehabilitation center linked to the ICU (Rehab-Hochzirl), we reported on treatment intensities and associations/relationships between late-occurring complications and patients’ trajectories. The functional outcome of these patients was quantified at Rehab-Hochzirl admission and discharge by using the mRS and the Barthel Index.

### Neurorehabilitation

After discharge from the neurological ICU, 87 (35%) out of 250 surviving patients were transferred to the neurological postacute unit Hochzirl. Neurorehabilitation in Hochzirl follows a multimodal concept. The stages of treatment extend from phase A, the acute phase, to phase E, in which social and professional reintegration takes place [16, 17]. Our postacute neurological department specializes in treating patients in phase B and early phase C. The goal-setting was adapted to the individual needs of the patients and therapy progress was evaluated in a standardized weekly meeting consisting of an interdisciplinary team of neurological physicians and therapists. According to local regulations, patients received a maximum of 3 h of therapy, consisting of units of 45 min, on working days. The permitted therapy curriculum was used, provided that the clinical condition of the patient allowed it. The interdisciplinary team enabled a variety of conventional therapies such as physiotherapy, occupational therapy, and speech therapy. In case of neurological deterioration, a detailed symptom-oriented investigation was performed. Liquor circulation disorders were diagnosed using cranial computed tomography scans and a “tap-test” in the course of a lumbar puncture. Epileptic seizures were treated with levetiracetam as first-line treatment.

### Statistical Analysis

The primary aim of the study was mRS improvement, with any decrease in the mRS (greater than one point). As a secondary end point, we defined good functional outcome after 1 year as mRS < 3. Continuous variables are reported as means ± standard deviations or as medians and interquartile ranges, as appropriate. The H&H scale and the modified Fisher scale were used as continuous variables in the analyses. Patients who died in the ICU, as well as patients with a discharge mRS score of 0 (*n* = 35), were excluded from the analyses. To identify explanatory variables and describe their relationship to the 3-month/1-year outcome, we have used generalized linear models with a binary logistic function and “Logit” as a link function.

To build our multivariate analysis model, we first identified in univariate analysis the associated factors that reached a *p* value < 0.1. These were introduced in the multivariate model and retained when significant (*p* < 0.05).

At the 12-month visit, 30% (*n* = 88) of patients were lost to follow-up. Missing outcome variables were imputed using the nearest neighbor matching (VIM version 6.1.0). K-Nearest Neighbor imputation is based on a variation of the Gower Distance for numerical, categorical, ordered, and semicontinuous variables. Graphical representations and analysis were performed with Prism 5 for Windows V5.01 (GraphPad Software, Inc, LA Jolla, CA; IBM-SPSS V24.0), Statistical Package for the Social Sciences (SPSS Inc, Armonk, NY) and SankeyMATIC (BETA; http://sankeymatic.com/).

## Results

### General Information

**Fig. 2:**
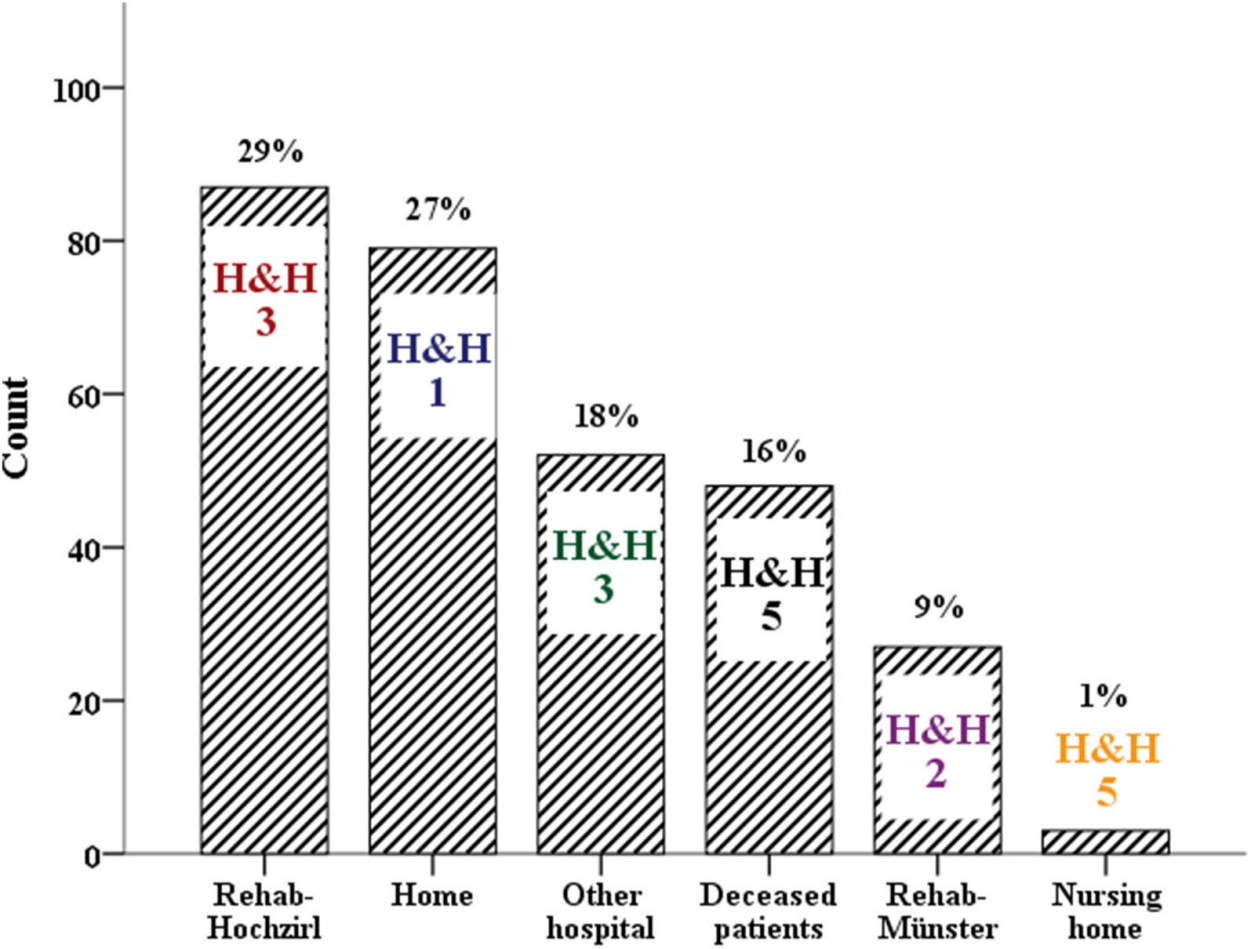
Distribution of ICU discharges. *H&H* Hunt and Hess scale, *ICU* intensive care unit

Out of 324 patients, 298 patients with spontaneous SAH were included in the final analysis. Detailed information on patients’ characteristics, hospital complications, and outcomes is given in Table 1. Patients were 57 ± 14 years old and stayed in the ICU for 16 (8–28) days. All severity grades were included, with a median H&H score of 2 (1–4) on ICU admission. At ICU discharge, the median mRS was 4 (1–5), and 16% (*n* = 48) of patients died during hospitalization. Out of the 250 surviving patients, 35% were transferred directly to Rehab-Hochzirl for neurorehabilitation (*n* = 87/250; mRS at ICU discharge 4 [3–5]), 11% to another rehabilitation center (*n* = 27/250; mRS 1 [1–3]), 1% to a nursing home due to severe disease without potential for rehabilitation (*n* = 3/250; mRS 5 [5]), 21% were transferred to their country of origin (*n* = 52/250; mRS 4 [2–5]), and 32% (*n* = 79/250; mRS 1 [0–1]) were discharged home (Fig. 2, Supplemental Fig. 1).

**Table 1:** Baseline characteristics, hospital complications, and outcomes of 298 patients with subarachnoid hemorrhage

Characteristics	All patients, *N* = 298 (100%)	Rehab-Hochzirl, *n* = 87 (29%)	Rehab-Münster, *n* = 27 (9%)	Home, *n* = 79 (27%)	Nursing home, *n* = 3 (1%)	Other hospital, *n* = 52 (18%)	Deceased patients, *n* = 48 (16%)
Baseline characteristics							
Age, mean ± SD (year)	57 ± 14	58 ± 13	56 ± 10	49 ± 12	66 ± 9	62 ± 13	62 ± 17
Female sex	182 (61)	64 (74)	20 (70)	31 (39)	2 (67)	34 (65)	32 (67)
H&H score ICU admission	2 (1–4)	3 (2–5)	2 (2–3)	1 (1–2)	5 (5–5)	3 (1–5)	5 (5–5)
GCS ICU admission	13 (3–15)	9 (3–14)	15 (8–15)	15 (15–15)	3 (3–3)	4 (3–15)	3 (3–3)
Clipping	73 (25)	40 (47)	7 (26)	6 (8)	2 (67)	9 (17)	7 (15)
Coiling	139 (47)	42 (49)	18 (67)	28 (35)	0 (0)	32 (62)	20 (42)
No intervention	85 (28)	4 (5)	2 (7)	45 (57)	1 (33)	11 (21)	21 (43)
Radiographic characteristics							
Aneurysm detected	229 (76)	83 (95)	25 (93)	34 (43)	2 (67)	43 (83)	40 (83)
Aneurysm > 10 mm	38 (13)	12 (14)	0 (0)	5 (6)	1 (33)	9 (17)	10 (22)
ICH present on admission	60 (29)	21 (24)	3 (11)	4 (5)	2 (67)	8 (15)	20 (42)
Hospital complications							
Pneumonia	121 (41)	51 (59)	8 (30)	9 (11)	3 (100)	33 (64)	16 (33)
Urinary tract infection	68 (23)	32 (37)	5 (19)	13 (17)	3 (100)	12 (23)	2 (4)
Ventriculitis	32 (11)	18 (21)	6 (22)	0 (0)	1 (33)	5 (10)	2 (4)
Hydrocephalus EVD	140 (47)	58 (67)	14 (52)	9 (11)	3 (100)	31 (60)	24 (50)
Vasospasm	129 (43)	57 (66)	10 (37)	20 (25)	2 (67)	27 (52)	12 (25)
DCI	50 (17)	21 (24)	5 (19)	5 (6)	1 (33)	9 (17)	8 (17)
Outcome characteristics							
ICU length of stay	16 (8–28)	28 (18–40)	16 (10–22)	10 (7–17)	74 (68–74)	17 (11–26)	4 (1–17)
mRS at ICU discharge	4 (1–5)	4 (3–5)	1 (1–3)	1 (0–1)	5 (5–5)	4 (2–5)	6 (6–6)
0	35 (12)	0 (0)	0 (0)	34 (43)	0 (0)	1 (2)	0 (0)
1	60 (20)	6 (7)	15 (56)	34 (43)	0 (0)	5 (10)	0 (0)
2	23 (8)	6 (7)	5 (17)	5 (6)	0 (0)	7 (14)	0 (0)
3	27 (9)	18 (21)	5 (17)	1 (1)	0 (0)	3 (6)	0 (0)
4	40 (13)	26 (30)	1 (4)	1 (1)	0 (0)	12 (23)	0 (0)
5	65 (22)	31 (36)	1 (4)	4 (5)	3 (100)	24 (46)	0 (0)
6	48 (16)	0 (0)	0 (0)	0 (0)	0 (0)	0 (0)	48 (100)

Data are given as mean ± SD, as median (IQR), or as counts (%)

*DCI* delayed cerebral ischemia, *EVD* external ventricular drain, *GCS* Glasgow Coma Scale, *H&H* Hunt and Hess, *ICH* intracerebral hemorrhage, *ICU* intensive care unit, *IQR* interquartile range, *mRS* modified Rankin Scale, *SD* standard deviation

### Functional outcome 3 and 12 months after bleeding

**Fig. 3:**
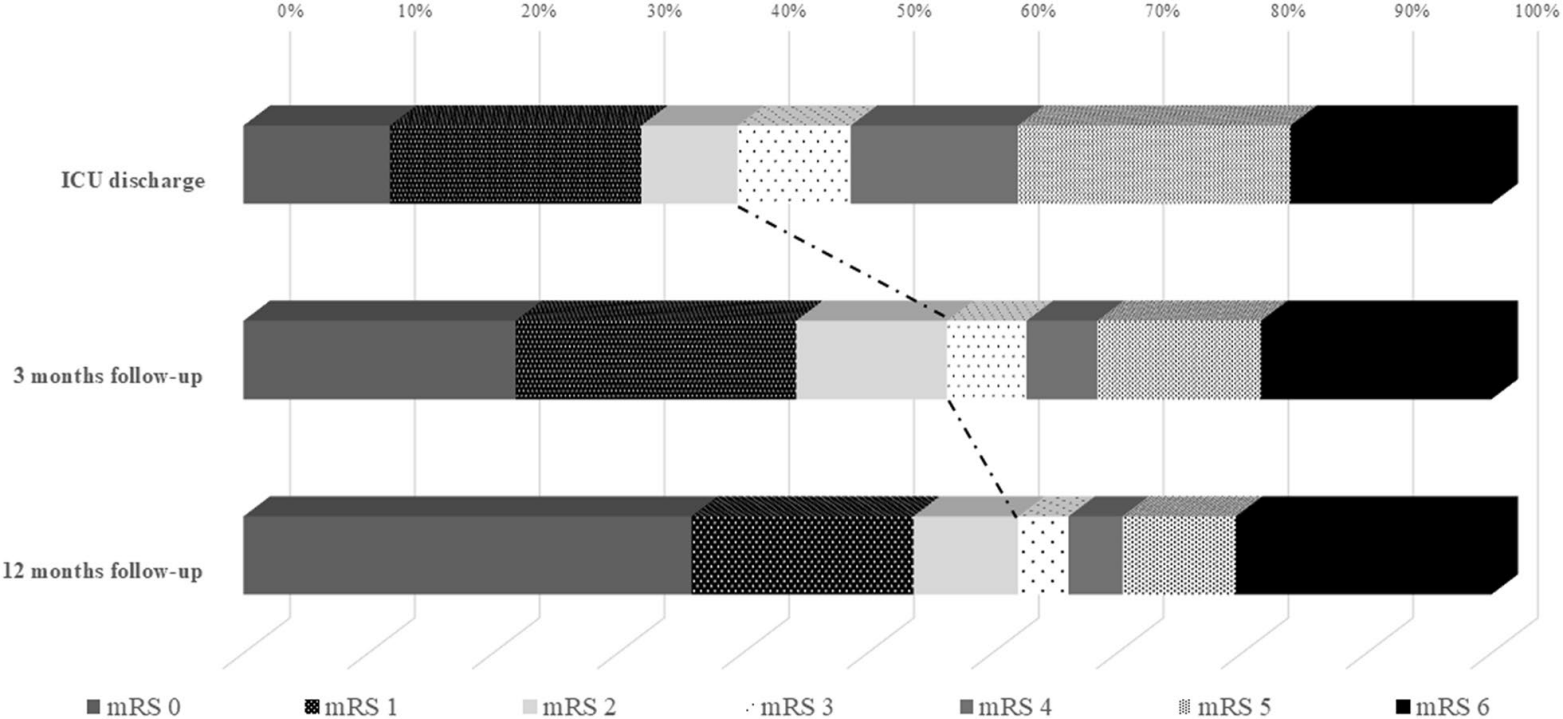
Count of patients per mRS category at ICU discharge, 3 months, and 12 months after SAH in 298 patients with SAH. The number of patients in each mRS category increased steadily from the time of acquisition through the time of discharge. *ICU* intensive care unit, *mRS* modified Rankin Scale, *SAH* subarachnoid hemorrhage

Out of all patients with the potential to improve (mRS ICU discharge 1–5, *n* = 215), functional outcome improved in 57% (*n* = 122/215) during the first 3 months after SAH. Additionally, 16% (*n* = 35/215) improved between 3 and 12 months, resulting in an overall improvement in 73% (*n* = 157/215) of patients. After 1 year, 60% (*n* = 179/250) of SAH survivors were functionally independent (mRS < 3). Worsening of functional outcome occurred in 6% (*n* = 16/250) of patients within 12 months (Fig. 3, Supplemental Fig. 2).

### Functional outcome after neurorehabilitation and intensity of therapy

Patients were admitted to Rehab-Hochzirl at median 38 (26–49) days after SAH, and presented with a median mRS of 4 (3–5) and a median Barthel Index of 30 (0–85) points. Medical complications during rehabilitation are listed in Table 2. Patients stayed in Rehab-Hochzirl for a median of 8.2 (4.2–19.0) weeks. Patients received a median of 3.1 (2.7–3.4) units of physical therapy per week. A total of 84 (97%) patients underwent occupational therapy, with a median of 2.7 (1.8–3.1) sessions per week. Seventy-four patients (85%) with speech or swallowing disorders of varying severity underwent speech therapy (1.49 [0.28–3.25] units per week). In 42/87 patients (48%), neurorehabilitation was assisted by robotic aids. Supplemental Table 1 provides an overview of the therapies applied during the rehabilitative stay in Rehab-Hochzirl. We found differences in terms of therapy intensity across mRS groups (mRS 0–2 vs. mRS 3–4 vs. mRS 5). Patients with higher mRS at Rehab-Hochzirl admission received more weeks of therapy (*p* < 0.001) as well as more (absolute minutes) of physical therapy, speech-language pathology, occupational therapy, and robotic assistive device therapy (*p* < 0.001, *p* < 0.001, *p* < 0.001, and *p* = 0.017, respectively).

**Table 2:** Clinical characteristics and complications at Rehab-Hochzirl admission and during the rehabilitation stay

Characteristic	Patients (*n* = 87)
Length of Rehab-Hochzirl stay (day)	42 (22–104)
CIP/CIM Rehab-Hochzirl admission	12 (14)
CIP/CIM Rehab-Hochzirl discharge	8 (9)
Dysphagia Rehab-Hochzirl admission	32 (37)
Dysphagia Rehab-Hochzirl discharge	19 (22)
Complications during Rehab-Hochzirl stay	
Pneumonia	5 (6)
Sepsis	2 (2)
Urinary tract infection	23 (26)
Organic brain disorder	4 (5)
Delayed aneurysm rebleeding	3 (3)
Gastrointestinal disorders	7 (8)
VP shunt infection	4 (5)
New VP shunt	5 (6)
Newly diagnosed hydrocephalus	6 (7)
Epileptic seizure	5 (6)
Ventriculitis	1 (1)
Multiple organ system failure	1 (1)

Data are given in median (IQR) and counts (%)

*CIM* critical illness myopathy, *CIP* critical illness polyneuropathy, *IQR* interquartile range, *VP* ventriculo-peritoneal

**Fig. 4:**
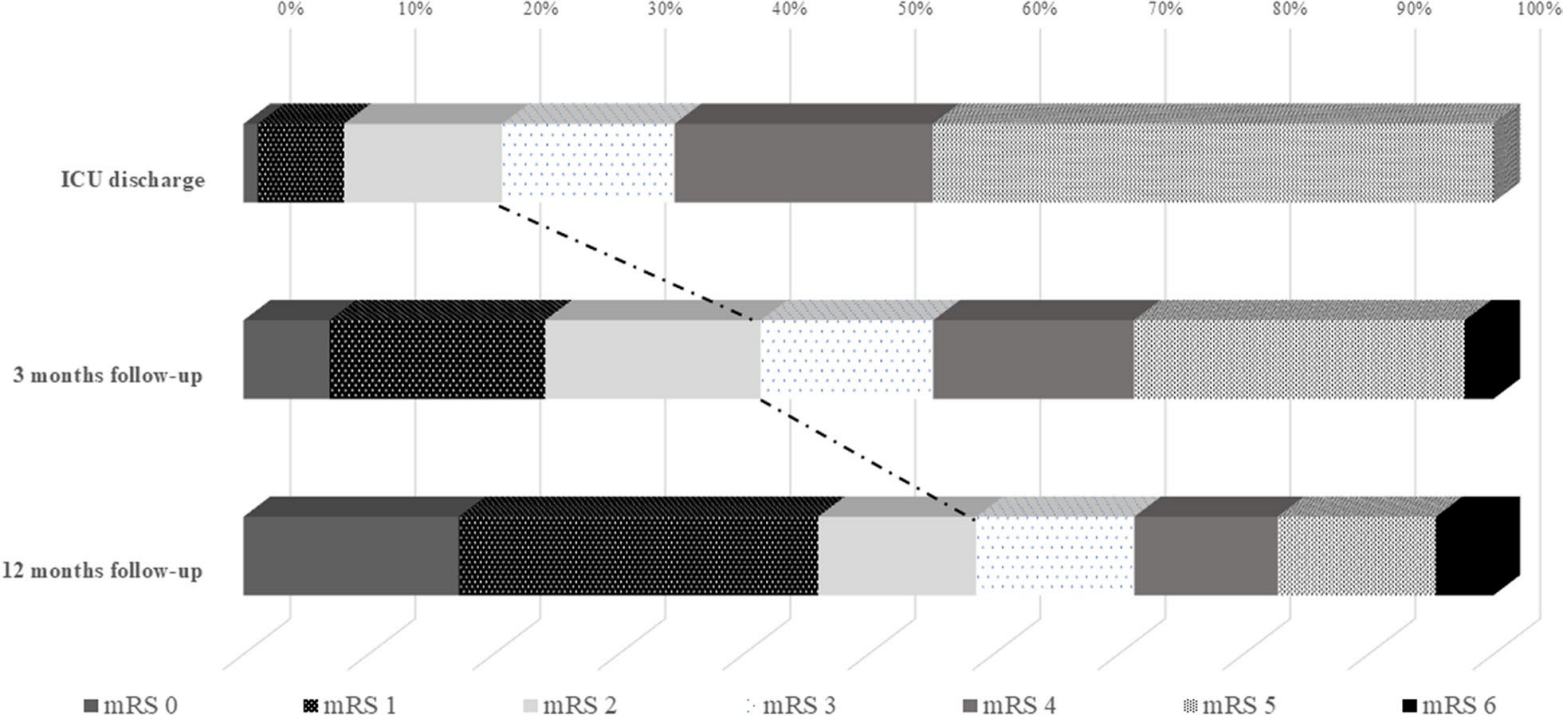
Subgroup analysis: count of patients per mRS category at ICU discharge, 3 months, and 12 months after subarachnoid hemorrhage in 87 Rehab-Hochzirl patients. The number of patients in each mRS category increased steadily from the time of acquisition through the time of discharge. *ICU* intensive care unit, *mRS* modified Rankin Scale

A total of 52/87 patients (60%) improved during inpatient neurorehabilitation, 32/87 patients (37%) remained stable, and 3/87 patients (3%) deteriorated, out of whom 2 patients died during the Rehab-Hochzirl stay (Fig. 4). Patients were discharged from Rehab-Hochzirl with a median mRS of 3 (2–5) and a median Barthel Index of 75 (11–100) points indicating a median improvement of ∆mRS = 1 point and ∆Barthel Index points = 45, respectively.

### Factors associated with improvement in functional outcome

In univariate analysis, younger age, a better clinical presentation on admission to the ICU and an uncomplicated clinical course in the ICU were significantly associated with functional improvement during the observation period of 12 months after SAH (Table 3).

**Table 3:** Univariate analysis of factors associated with improvement of the mRS score during the observation period of 12 months after SAH

Parameter	OR	95% CI	Univariate *p* value
ICU discharge mRS	0.65	0.52–0.80	**< 0.001**
Age (year)	0.96	0.94–0.98	**< 0.001**
Age > 60 years	0.35	0.19–0.66	**< 0.001**
ICU admission Hunt & Hess score	0.75	0.61–0.92	**0.005**
ICU admission modified Fisher score	0.77	0.57–1.05	0.095
ICU admission GCS score	1.09	1.03–1.15	**0.002**
ICU admission GCS score < 15	0.46	0.24–0.90	**0.024**
Hydrocephalus requiring EVD	0.64	0.35–1.19	0.161
Presence of an aneurysm	0.49	0.19–1.23	0.129
Intubation	0.41	0.15–1.13	0.084
Intubated days	0.95	0.93–0.98	**0.002**
Female sex	0.39	0.19–0.78	**0.009**
Ventriculitis	1.26	0.51–3.11	0.617
Pneumonia	0.49	0.26–0.89	**0.021**
Urinary tract infection	0.51	0.27–0.96	**0.037**
Vasospasm	0.69	0.37–1.27	0.229
Delayed cerebral ischemia	0.69	0.33–1.42	0.313

Data are given in median (IQR) and counts (%)

Statistically significant values are given in bold

*CI* confidence interval, *EVD* external ventricular drain, *GCS* Glasgow Coma Scale, *ICU* intensive care unit, *IQR* interquartile range, *mRS* modified Rankin Scale, *OR* odds ratio

In multivariate analysis, a lower H&H score at ICU admission (adjusted odds ratio [adjOR] 0.79, 95% confidence interval [CI] 0.64–0.98, *p* = 0.033), younger age (per year, adjOR 0.96, 95% CI 0.94–0.99, *p* = 0.002), a lower mRS at ICU discharge (adjOR 0.74, 95% CI 0.56–0.97, *p* = 0.031), fewer days on mechanical ventilation (per day, adjOR 0.96, 95% CI 0.93–0.99, *p* = 0.041) and male sex (adjOR 0.45, 95% CI 0.22–0.95, *p* = 0.036) were independently associated with functional recovery, corrected for age, H&H score, and aneurysm status (Table 4).

**Table 4:** Factors associated with improvement in functional outcome in all patients (n = 298)^a^

Parameter	adjOR	95% CI	*p* value
mRS at ICU discharge	0.74	0.56–0.97	**0.031**
Days on mechanical ventilation, per day	0.96	0.93–0.99	**0.041**
Male sex	0.45	0.22–0.95	**0.036**
Age, per year	0.96	0.94–0.99	**0.002**
Hunt & Hess score	0.79	0.64–0.98	**0.033**

Subgroup analysis: 87 Rehab-Hochzirl patients^b^	adjOR	95% CI	*p* value
Dysphagia on Rehab-Hochzirl admission	0.33	0.11–0.97	**0.44**
Age, per year	0.96	0.92–0.99	**0.031**

Statistically significant values are given in bold

*adjOR* adjusted odds ratio, *CI* confidence interval, *ICU* intensive care unit, *mRS* modified Rankin Scale, *SAH* subarachnoid hemorrhage

^a^During the observation period of 12 months after SAH, adjusted for age, Hunt & Hess score, and aneurysm state

^b^During inpatient neurorehabilitation adjusted for age, Hunt & Hess score, and aneurysm state

In the subset of patients transferred for inpatient neurorehabilitation to Rehab-Hochzirl, younger age (per year, adjOR 0.96, 95% CI 0.92–0.99, *p* = 0.031) was independently associated with improvement in the mRS during inpatient neurorehabilitation, even after adjusting for H&H Grade on admission and aneurysm state. Moreover, patients with dysphagia on Rehab-Hochzirl admission (adjOR 0.33, 95% CI 0.11–0.97, *p* = 0.044) were independently associated with a lack of improvement or deterioration in the mRS, corrected for H&H, age, and aneurysm status (Table 4). During the rehabilitative stay in Rehab-Hochzirl, patients who did not improve were more often diagnosed with urinary tract infections (OR 3.4, 95% CI 1.3–9.21, *p* = 0.015).

Patients who did not improve stayed in Rehab-Hochzirl for a median of 52 (26–123) days compared with 36 (22–90) days in patients with mRS improvement (*p* = 0.075). Importantly, patients with a consistent mRS score had the same duration of physiotherapy (*p* = 0.277), speech therapy (*p* = 0.415), occupational therapy (*p* = 0.286), or robotic therapy (*p* = 0.148) as patients with an improvement.

## Discussion

The main findings of this study are that improvement in functional outcome is particularly evident during the first 3 months after SAH, underlining the importance of early neurorehabilitation. However, our results also indicate that a substantial number of patients (16%) still recovers from disability thereafter, which supports longitudinal care and follow-up in patients with SAH. In patients with close monitoring during specialized inpatient neurorehabilitation, we observed functional improvement despite evidence for (severe) disability on admission.

Long-term care and functional outcome after ischemic stroke have been reported in several studies [8, 18, 19]. Interestingly, clinical trajectories of patients with SAH over a prolonged time period have rarely been reported so far [20]. In a meta-analysis including 33 articles derived from 39 prospective population-based studies (19 countries spanning 30 years, 8739 patients, mean age 62 years) 55% of patients with SAH recovered to an independency in daily activities after 1–12 months, whereas 19% remained dependent and 26% died [3, 20]. In a study conducted by Wilson et al. [21], 61% of patients with SAH showed an improvement in mRS in the first 6 months and another 18% after 12 months. This is consistent with our findings, in which we found an improvement of functional outcome in the first 3 months in 57% with an additional 16% of patients improving within the first year post bleeding, resulting in a good functional outcome in 60% of patients. Recovery after SAH is a long-term and arduous process. In most studies, clinical improvement often does not occur until the end of follow-up [21], as functional outcome is mostly evaluated at a single time points soon after hospital discharge (3 or 6 months after ICU discharge).

Our data suggest that a considerable number of patients with SAH are severely disabled after ICU discharge (44%), which is an even higher prevalence rate than in patients with intracerebral hemorrhage or traumatic brain injury [22]. The modest functional outcome in our patient cohort at ICU discharge is consistent with recent findings in patients with SAH (mRS 4 [1–5]) [20, 21]. Importantly, these patients improved significantly during specialized neurorehabilitation including physical therapy, occupational therapy, speech therapy, and neurorehabilitation assisted by robotic aids, based on the individual patient needs.

There are several reasons why functional improvement may occur in neurocritical care patients even after several months. The brain has the ability to respond as a dynamic system to external influences and to establish new circuitry. This brain plasticity makes it possible for patients to recover clinically from severe cerebral hemorrhage. We found a lower initial severity, younger age, a lower mRS Score at ICU discharge, shorter days on mechanical ventilation, and male sex as independent factors for functional improvement. This corresponds to previous reports and may be explained by a higher potential for recovery in young patients with less severe injury patterns [23, 24]. Male sex was also associated with an improvement in functional outcome in this study, which may be owed to the lower age (men: 56 [47–67] years vs. women: 59 [48–68] years) on the one hand and a to lower initial disease severity on the other hand (H&H men: 2 [1–4] vs. women: 3 [2–5]). Interestingly, we found that functional improvement was common in patients with a high degree of disability who were transferred to a nearby neurorehabilitation facility (Rehab-Hochzirl). Overall, 60% improved due to or with neurorehabilitation with a median improvement of ∆ mRS = 1 and ∆ Barthel Index points = 45 during the rehabilitation period of 8.2 (4.2–19.0) weeks, respectively. Similarly, younger age and a lower H&H score on ICU admission seem to be indicators in favor of patient improvement during inpatient neurorehabilitation, which may help in the identification of a subgroup with high potential for recovery. Although the intensity of rehabilitation did not differ, one of the patient subgroups failed to improve. It is well known that swallowing disorders after ischemic stroke, as well as after SAH, are associated with poor functional outcome [25–27]. Besides elevated age and disease severity, evidence for dysphagia was a strong predictor for failure of recovery in our cohort. The importance of persistent swallowing disorders after ischemic stroke received more attention in the past few years, with new innovations like pharyngeal stimulation as an effective intervention to improve outcome and recovery [28]. Interestingly, occurrence of infections during neurorehabilitation were associated with failure of recovery. It is well known that infectious complications during ICU stay are associated with prolonged ICU stay and poor outcome [29, 30]. Our results indicate that close monitoring and infection surveillance is important beyond the acute phase.

Although neurorehabilitation is recommended (American Heart Association guidelines, evidence, recommendation Class 1A), its effects are not well studied and a large variability exists due to local infrastructure, socioeconomic disparities and the lack of availability in several parts of the world [8, 31]. It is recommended to perform rehabilitation exercises on a regular basis to activate specific brain areas and enhance interneuronal networks [32, 33]. Although therapy frequency and the duration of single sessions depend on local regulations, there is strong evidence suggesting that intensifying and repeating interventions enhances functional recovery [34].

The heterogenous clinical presentation at hospital discharge after SAH argues for an individualized neurorehabilitation approach in specialized centers, which renders clinical trials challenging. With the lack of a consensus on the optimal modality and the optimal time period of rehabilitation, current concepts vary across centers and are mainly based on the local expertise and experience. In recent years, substantial progress in the development of evidence-based therapy principles for stroke patients has been made, which may be translated to patients with SAH [32]. Although the ideal time to start rehabilitation is uncertain, early rehabilitation supports remodulation processes, which occur early after SAH as suggested by animal models [35]. It is important to mention that neurorehabilitation in our ICU was initiated as early as possible, stratified by potential side effects and the clinical condition of the patient. Early initiation of neurorehabilitation may be important, given that we observed a higher degree of improvement within the first 3 months after SAH, in accordance with other acute brain injuries [36].

There are some limitations that merit consideration. Due to the single-center nature of our study, our data may not be generalizable to other settings or other parts of the world with decreased access to neurorehabilitation. At the 12-month visit, 30% (*n* = 88) of patients were lost to follow-up. The high dropout rate is mostly explained by the high number of patients (*n* = 52) visiting the referral area of our hospital as tourists. This can be explained by the fact that the province (Tyrol) in which the hospital is located is a touristic hotspot. Rough estimations approximate 5 million visitors due to tourism per year [37]. As previously done in large studies, such as the Center Traumatic Brain Injury study [38], missing outcome variables were imputed using the nearest neighbor matching. The imputation process did not change the distribution of the outcomes: there was no difference in relation to mRS discharge in patients lost to follow-up and those with known follow-up (*p* = 0.072). Furthermore, we used functional outcome as primary endpoint which is known to be imprecise to detect the full range of morbidities after SAH. Assessment of quality of life, return to previous work capacity and a detailed neuropsychological evaluation are vital to enhance and understand clinical trajectories after SAH. In addition, we do not have a comparison group because in our hospital every patient with potential for improvement receives access to neurological rehabilitation. Therefore, a comparison group to study the effects of intensive neurorehabilitation in patients with and without intensive rehabilitation would not be ethically justifiable. In this study, a nonunique approach to the follow-up assessment was done. However, Savio et al. [39] and Janssen et al. [40] reported sufficient reliability of telephone assessment versus face-to-face assessment. In this context, we see the type of data collection as of secondary importance. In addition, the period of one year may be too short to capture further improvement after SAH. Further prospective studies with longer follow-ups are needed. Finally, we cannot proof causality (e.g., effect of neurorehabilitation) based on the retrospective analysis of prospectively collected data.

## Conclusions

Clinical trajectories over a 1-year period after SAH suggest that there is a huge potential for improvement in a substantial number of patients. Continuation of care with regular follow-ups even beyond 1 year may improve our knowledge on long-term outcome after SAH. Our data further suggest that neurorehabilitation may be of benefit even for patients with substantial deficits after the acute phase. Future studies with additional investigations to quantify disease burden on quality of life, cognition as well as social and psychological end points, are needed.

## Supplementary Information

Below is the link to the electronic supplementary material.

Supplementary file1 (JPG 542 kb)

Supplementary file2 (JPG 530 kb)

Supplementary file3 (DOCX 14 kb)
