# Supplementary figures and images for: Long-Term Clinical Trajectory of Patients with Subarachnoid Hemorrhage: Linking Acute Care and Neurorehabilitation
Source: Neurocrit Care. 2022 Aug 12;38(1):138–48. doi: 10.1007/s12028-022-01572-6 (PMC9935743; doi:10.1007/s12028-022-01572-6)

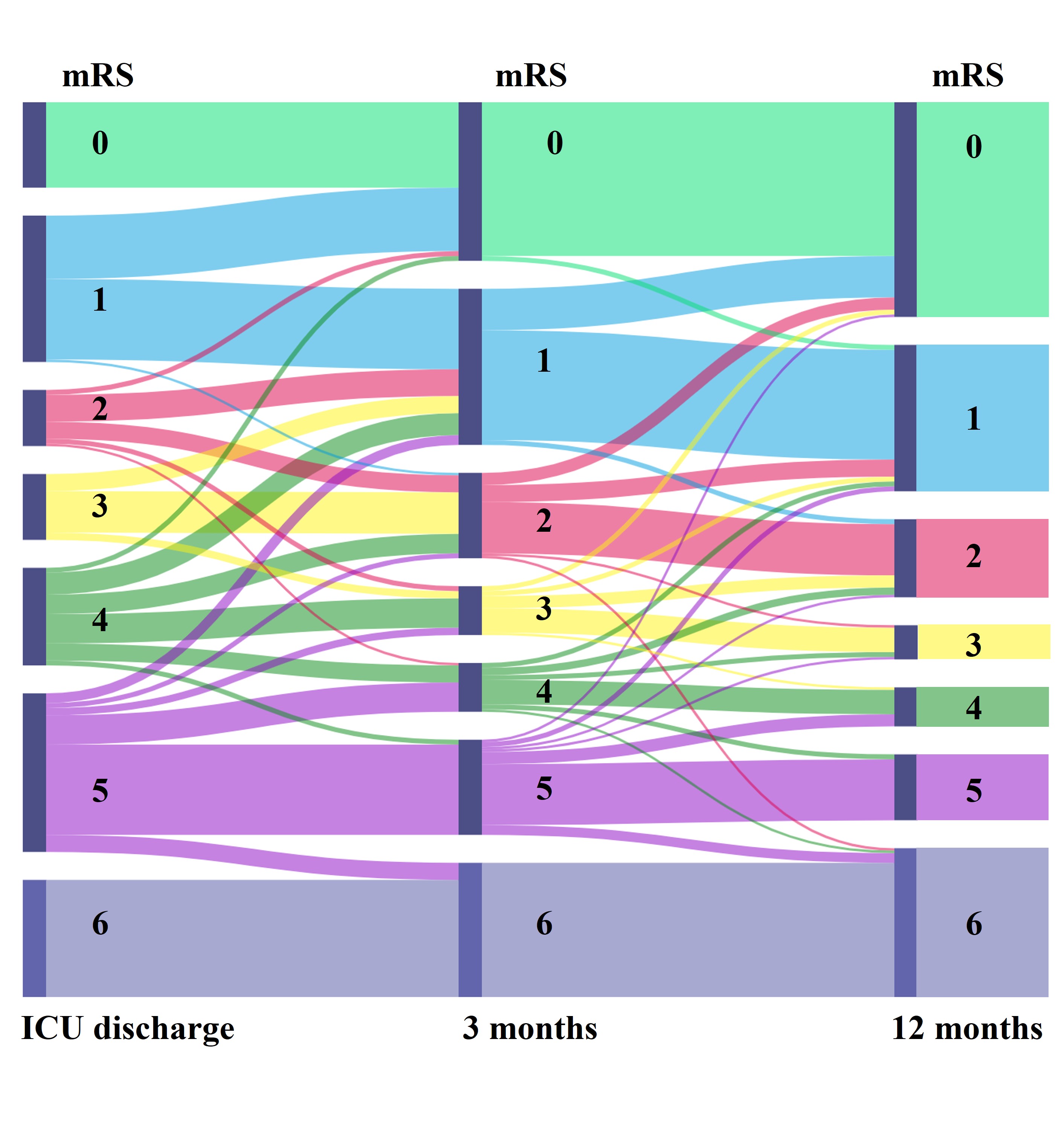

Supplement: Supplementary file 1 — Supplementary file1 (JPG 542 kb) [file 12028_2022_1572_MOESM1_ESM.jpg]

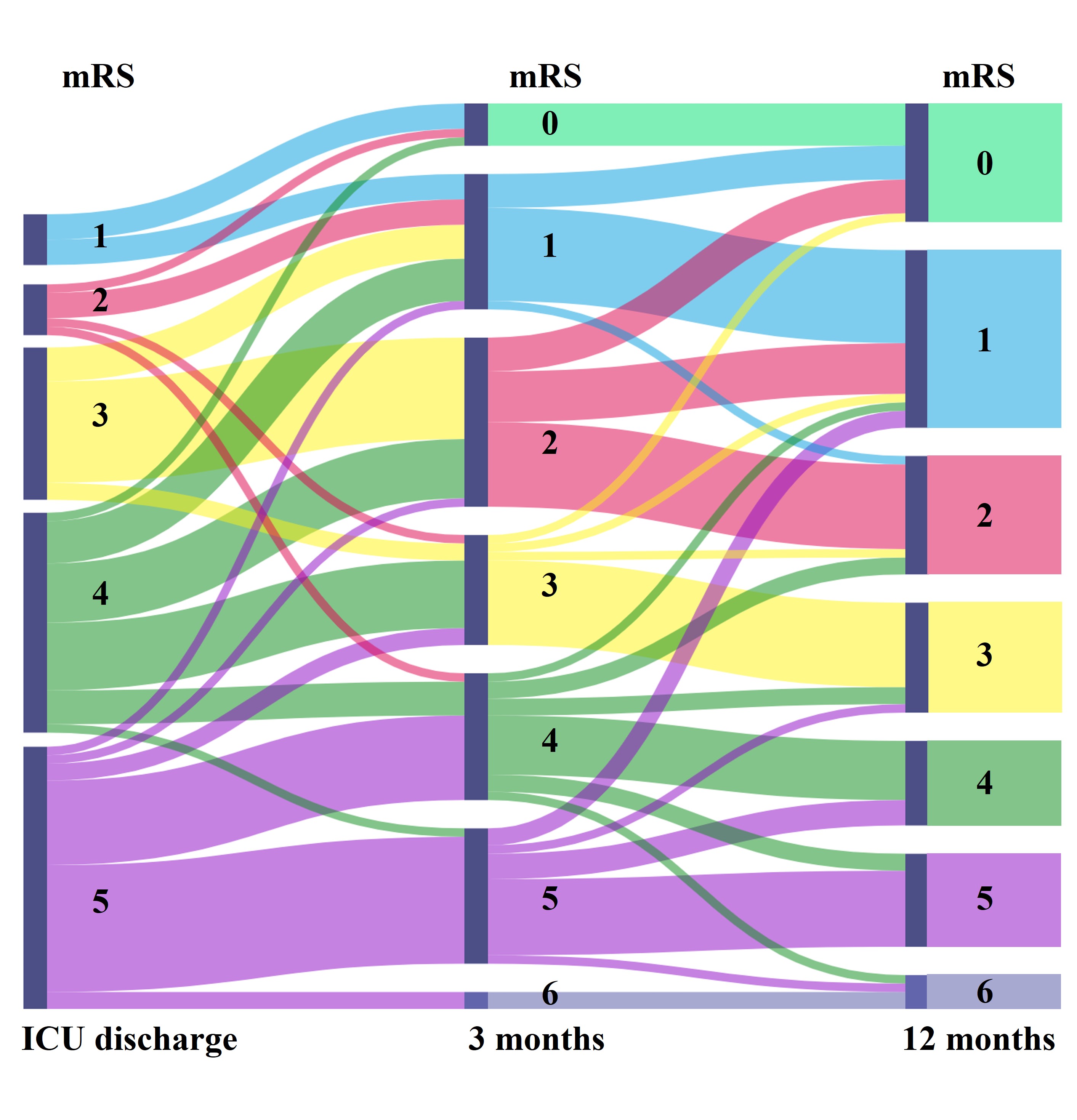

Supplement: Supplementary file 2 — Supplementary file2 (JPG 530 kb) [file 12028_2022_1572_MOESM2_ESM.jpg]
