# Supplementary material for: Long-Term Clinical Trajectory of Patients with Subarachnoid Hemorrhage: Linking Acute Care and Neurorehabilitation
Source: Neurocrit Care. 2022 Aug 12;38(1):138–48. doi: 10.1007/s12028-022-01572-6 (PMC9935743; doi:10.1007/s12028-022-01572-6)
Supplement: Supplementary file 3 — Supplementary file3 (DOCX 14 kb) [file 12028_2022_1572_MOESM3_ESM.docx]

|  | n (%) or median [IQR] |
| --- | --- |
| Physiotherapy | 87 (100) |
| Physiotherapy, minutes | 1038 [90-7470] |
| Physiotherapy, units | 26 [2-143] |
| Physiotherapy units / week | 3.11 [1-4] |
| Speech therapy | 74 (85) |
| Speech therapy, minutes | 825 [15-7440] |
| Speech therapy, units | 18 [1-163] |
| Speech therapy units / week | 1.49 [0-5] |
| Occupational therapy | 84 (97) |
| Occupational therapy minutes | 915 [30-5595] |
| Occupational therapy units | 21.5 [1-124] |
| Occupational therapy units / week | 2.74 [0-13] |
| Robotic therapy | 42 (48) |
| Robotic therapy, minutes | 1330 [20-5155] |
| Robotic therapy units | 29 [1-159] |
| Robotic therapy units / week | 0 [0-4] |
| Weeks of therapy (5 days) | 8.2 [1-41] |

**Supplemental Table 1. Intensity of Neurorehabilitation – Overview**

Data are given in median (IQR) and counts (%).
